# Supplementary material for: Open Knowledge about Slaughter on the Internet—A Case Study on Controversies
Source: Animals (Basel). 2017 Dec 18;7(12):101. doi: 10.3390/ani7120101 (PMC5742795; doi:10.3390/ani7120101)
Supplement: Supplementary file 1 [file animals-07-00101-s001.pdf]

Information before Workshop

Here is some information for the participants in the workshop on the 20th pf January relating to the research project "Quality assurance of DISA." The workshop is structured as a focus group interview and led by researcher Magnus Ljung, and based on the educational material about slaughter of animals, which is openly available on disa.slu.se

Welcome!

Time: Wednesday 20th of January at 10:00 to 12:00.

Location: We will conduct the workshop via video link. It means that you choose the city closest to you: 1) Department of Animal Environment and Health, Swedish University of Agricultural Sciences, Skara. 2) Department of Animal Environment and Health, Swedish University of Agricultural Sciences, Uppsala, 3) Department of Applied IT, Gothenburg University, Gothenburg.

Contact: Anne Algers, Lotta Berg or Magnus Ljung.

Form: Focus group interviews are structured group discussions where the moderator allows the participants to influence the content. Through the use of a number of statements, the moderator steers the discussion so that a number of specific topics are discussed. These statements are ideas generated during the project, and where your reactions and opinions are central.

Objectives: To assure the quality of the process of development and use of the open educational resource DISA in order to ensure that DISA a) is inclusive, b) can be used for managing controversies, c) can increase awareness and knowledge about slaughter and killing, d) is current and adapted to a changing world, e) is relevant for both employees at slaughterhouses and for students and the public. We will also discuss if a new English version and a new interface can enhance a) inclusion b) conflict management c) awareness and knowledge about slaughter and d) accuracy and relevance. We also want a more general discussion about sustainable development and open and social learning in a controversial area.

Results: We expect that the meeting will generate added value for all invited. This means not only that you get the opportunity to raise issues and perspectives in relation to the DISA material. We also hope for a more general discussion about learning and communication of knowledge in controversial areas of societal interest. We also expect that it is valuable for the participants to meet each other and exchange experiences!

Come as you are: You are invited because you and the other participants together form a group with broad representation of different interests and perspectives. Each of you is selected based on your knowledge and experience. We do not make any demands on you to anchor your thoughts in your organization in advance or in retrospect, it's your personal opinion we are looking for. No special preparation is required before the workshop. Your involvement will determine the direction and outcome of the focus group. Your statements will be treated anonymously and only type of organization will be evident from the report/article.

Contents: Themes that we want to discuss are for example, questions about inclusiveness in DISA and other open educational resources, the extent to which DISA is a cause for conflict, how we can increase positive contacts between academia, the slaughterhouses and the general public, how DISA can be used to capture public perceptions of animal welfare at slaughter and killing, strengths and weaknesses with a relatively open system, how we achieve and maintain transparency, how it is related to sustainable

development. We try to take a holistic approach, and therefore we will switch between large and small in the debate.

About the project "Animal welfare at slaughter - an open and social learning process": We do research within the field of learning and ICT, animal welfare and environmental communication. The focus group is thus not primarily about the content, but more about how different actors relate to a specific knowledge (in this case animal welfare at slaughter and killing), and to other stakeholders. Focus is on the relationship between academia, authorities, students, slaughterhouses and society on the individual and organizational level.

We want to look at the potential for increased transparency in a controversial topic and what the consequences may be. What is the potential for change in this context and what are the risks?

As part of this project we organize two focus group interviews. The aim is to gather existing perspectives and ideas and develop criteria and models that have relevance both for further research and for those who work with DISA.
